# Nonlinear Impedance of Whole Cells Near an Electrode as a Probe of Mitochondrial Activity

**DOI:** 10.3390/bios1020046

**Published:** 2011-04-11

**Authors:** Akilan Palanisami, George T. Mercier, Jie Fang, John H. Miller

**Affiliations:** Department of Physics and Texas Center for Superconductivity, University of Houston, Houston, TX 77204, USA; E-Mails: gmercier@central.uh.edu (G.T.M.); Jie.Fang@mail.uh.edu (J.F.); jhmiller@uh.edu (J.H.M.)

**Keywords:** nonlinear dielectric spectroscopy, bioimpedance, mitochondria, fermentation

## Abstract

By simultaneously measuring the bulk media and electrode interface voltages of a yeast (*Saccharomyces cerevisiae*) suspension subjected to an AC voltage, a yeast-dependent nonlinear response was found only near the current injection electrodes. Computer simulation of yeast near a current injection electrode found an enhanced voltage drop across the yeast near the electrode due to slowed charging of the electrode interfacial capacitance. This voltage drop is sufficient to induce conformation change in membrane proteins. Disruption of the mitochondrial electron transport chain is found to significantly change the measured nonlinear current response, suggesting nonlinear impedance can be used as a non-invasive probe of cellular metabolic activity.

## 1. Introduction

The use of capacitance biosensors has a number of advantages, such as being label-free, simple and easily integrated into other multiplexed or miniaturized formats. Such sensors have generated considerable interest as detectors of biological analytes [1,2,3,4] and also for studying protein regulation in whole cells [5,6,7]. Low frequency electric fields are preferentially dropped across cell membranes, leading to amplified electric fields across membrane proteins [8]. As many membrane proteins are electrogenic in nature (such as membrane ion pumps), external electric fields can induce protein activity [9,10]. In principle, this coupling can be detected from the electrical behavior of the membrane protein. However, due to the small size of the cells and the interfacial electrode capacitance (across which much of the applied voltage is dropped at low frequencies), large, pulsed voltages are typically used to apply sufficient voltage across the cell or vesicle to induce detectable protein activity. As the applied voltages are too large for convenient electrical detection, the protein activity must be monitored by a separate chemical assay, which is slow and inconvenient with regard to clinical applications. One method to avoid the large voltage drop associated with the electrode capacitance is to use higher frequency excitation (~100 kHz). However, the time scale of electrogenic membrane protein activity is typically around 500 μs [11,12,13], which is too slow to be excited by such high frequency excitation.

Surprisingly, low voltage, lower frequency (~100 Hz) sinusoidal excitation has been found to generate harmonics in whole cells, but the origin of the harmonics and the connection with membrane protein activity has not been well-elucidated [7,14,15,16]. We have previously reported that 4-probe nonlinear dielectric spectroscopy is sensitive to the metabolic state of budding yeast [7], but this previous work did not explore if the harmonics originated from the bulk of the suspension [17] or from the electrode interfacial region [16]. This information is important for understanding the relationship between metabolism and the applied electric field. In addition, traditional nonlinear dielectric spectroscopy only looks at the frequency response of the system. While convenient, additional information useful for physical interpretation of the harmonics may be available in the time-domain response. However, the nonlinear response is typically masked by the large linear response, making time-domain analysis a difficult endeavor.

Here we examine if budding yeast (*Saccharomyces cerevisiae*) suspension produces harmonics near the electrode interfacial region or in the bulk of the suspension by redesigning the traditional 4 probe dielectric spectroscopy setup. We also use the additional information obtained from this setup to subtract out the linear component of the response, allowing direct visualization of the nonlinear response in the time domain. This behavior is then correlated with the aerobic respiration of the budding yeast suspension.

## 2. Experimental Section

Respiration-competent (ρ+) strain D273-10B [18] and deficient strain (ρ−) DS400/A12 [19] were prepared and oxygen consumption monitored as described [7]. Experiments were performed using a 2 mL suspension volume at a cellular concentration of 40 million cells/mL (~1% v/v). The suspension was mixed with a magnetic stir-bar during experiments.

**Figure 1:**
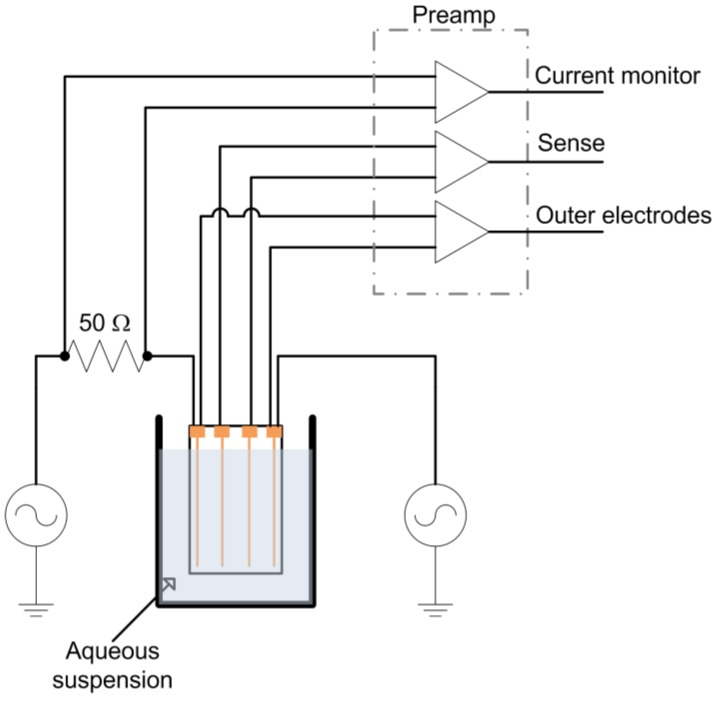
Experimental schematic.

A tetrapolar electrode configuration consisting of four, 100 μm diameter gold wires (99.99% pure) spaced 2 mm apart mounted on a glass microscope slide with epoxy adhesive at either end was constructed. This electrode was immersed in a yeast suspension to a depth of 1 cm during measurements (Figure 1). The outer wires were used as current injection electrodes and were connected in series to a 50 Ω metal film resistor (used for current measurement) and a Stanford Research Systems (SRS, Sunnyvale, CA) DS-360 Ultra Low Distortion Function Generator. The voltage across the series resistor (current monitor), outer current injection electrodes, and inner wire (sense) electrodes were monitored with INA121 preamplifiers (Texas Instruments, Dallas, TX), which were chosen due to their combination of linearity, high input impedance and low input bias current. The preamplifiers were all fixed at unity gain to avoid gain dependent phase variation. The preamplifier outputs were passed through AAF-2 Butterworth filters (f_c_ = 200 kHz, Alligator Technology, Costa Mesa, CA) before data acquisition at 250 kSamples/s/ch with a USB-6251 data acquisition card (National Instruments, Austin, TX). Data was acquired for 200 ms every 2 min. The function generator provided a 1 kHz, 4 V peak to peak (Vpp) sine wave. Subsequent data analysis was performed using DIAdem software (National Instruments).

## 3. Results and Discussion

Budding yeast was selected as a model for studying metabolic processes due to its well characterized mitochondrial biology. In addition, being a facultative anaerobe, yeast can metabolize substrates both aerobically and by fermentation. This characteristic allows yeast mutants which cannot perform aerobic respiration to survive. In particular, deletions of portions of mDNA (ρ−) result in mitochondria which lack proteins essential for aerobic respiration, forcing the yeast into existing entirely on fermentation [20]. In contrast, yeast with intact mDNA (ρ+) grown in a well oxygenated environment (as was done here) will use aerobic respiration primarily (until O_2_ is exhausted). These characteristics allow comparison of the effects of fermentation *versus* aerobic respiration on the nonlinear response. To avoid fluctuations in yeast metabolism, the D273-10B strain was chosen for its normal cytochrome content and resistance to glucose repression [18,20,21].

**Figure 2:**
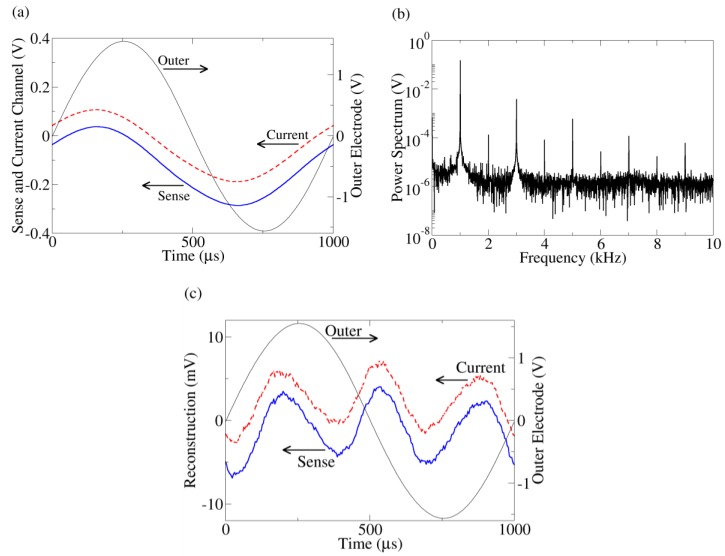
(**a**) Typical time series data from the outer electrode, current monitor, and sense channels. The current (dashed) and sense (solid) time traces are offset for clarity. Because of the symmetric probe configuration, when the outer electrode voltage is positive, yeast near one outer electrode will be hyperpolarized while, simultaneously, yeast touching the other outer electrode will be depolarized. Thus, both populations of yeast contribute to the response even though the applied voltage is positive (or negative). **(b)** Power spectrum of the current channel. The other harmonic peaks indicate distortion from a sine wave. The power spectrum of the sense channel has similar behavior. **(c) **Reconstruction of current (dashed) and sense (solid) channels after fundamental peak removal together with the outer electrode voltage. The reconstructed waveforms are offset for clarity.

Figure 2(a) shows a typical time trace of the series resistor (current), inner sense electrode (bulk), and outer electrode voltages from a yeast suspension measurement. The voltage across the outer (current injection) electrodes is the sum of the voltage drops across the bulk suspension and current injection interface, whereas the inner electrodes are proportional to the voltage drop across the bulk suspension only. Despite the pure sine wave produced by the function generator, a slight distortion is apparent in the sense and current channels. This distortion is clearly apparent after taking a Fourier transform of the data and examining the frequency spectrum of the data (Figure 2(b)). More insight can be gained by viewing the distortion in the time domain. To do this, the fundamental peaks (992.5 Hz–1,007.5 Hz) of the Fourier transform are zeroed and the waveforms reconstructed using the inverse Fourier transform (Figure 2(c)), leaving a “sense distortion” and a “current distortion”.

These reconstructed time traces can be used to determine the origin (bulk suspension or interface) of the nonlinearity. Kirchoff’s voltage law mandates the sum of all voltage distortions in the circuit must equal zero (this is true even for capacitive circuits). For this to occur, the voltage distortion in the series resistor must be anti-correlated (*i.e*., 180° phase shift) with the distortion in either the bulk or the interface. From Figure 2(c), we see the current distortion is almost perfectly correlated with the sense distortion, meaning the bulk voltage drop merely follows the current like a resistor. Thus, the nonlinearity must originate near the current injection interface, consistent with the electrode charging simulation. This technique of using anti-correlation to determine the location of nonlinearity has been established in another solid state system [22].

**Figure 3:**
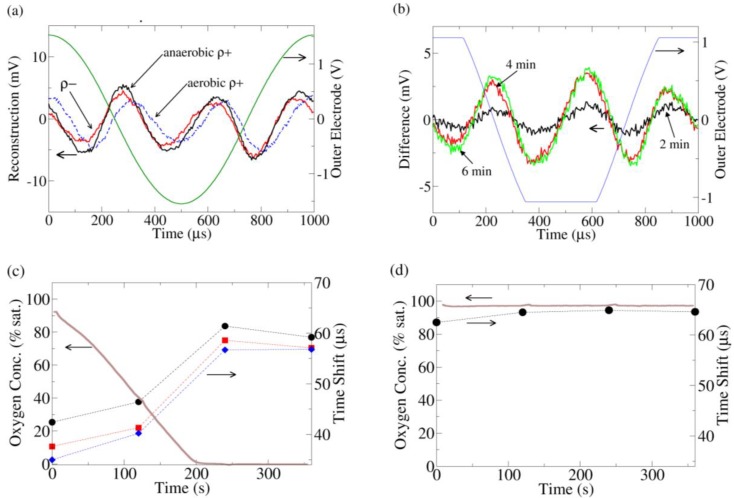
**(a)** Current distortion reconstruction from ρ+ (dashed) and ρ− yeast under O_2_ saturated conditions (data taken immediately after insertion of yeast into O_2_ saturated media) and ρ+ under O_2_ depleted conditions (data taken 6 min after insertion of yeast into media). The O_2_ saturated ρ+ data is clearly phase shifted to the right. **(b)** The change in the current distortion of ρ+ yeast as a function of time. The reconstruction at 0 min (100% O_2_ saturation) is used as a baseline subtraction. At 3.5 min, all the O_2_ has been consumed by the yeast. To increase the resolution of these data, the gain was increased on the analog to digital converter, leading to clipping of the recorded outer electrode voltage; thus the voltage sine wave across the outer electrodes appears clipped in the figure. **(c)** The time shift of the peak of the 3rd harmonic current distortion as compared to the peak of the fundamental outer electrode voltage (peaks are near time 0 in (a)) for 3 replicates of ρ+ yeast. As the O_2_ is consumed, the 3rd harmonic advances with respect to the fundamental. **(d)** Similar to (c) except the media now contains 10 μM antimycin. The time shift of the antimycin treated ρ+ yeast is similar to the ρ+ yeast in the anaerobic (fermenting) condition in (c).

A biological component to the nonlinearity can be seen by comparing the current distortion response from ρ+ and ρ- yeast in oxygen saturated media (Figure 3(a)). The distortion response of the ρ− yeast is advanced relative to the ρ+ strain. As the media was identical in both experiments, the difference must come from the yeast. Furthermore, when the chamber is sealed and the ρ+ yeast are allowed to consume all the oxygen, the ρ+ current distortion advances to the same phase as the ρ− current distortion. 

The change in ρ+ current distortion was also monitored in time (Figure 3(b)), using the current distortion from the start of the experiment (100% O_2_ saturation) as a baseline subtraction. At 3.5 min, all the O_2_ in the media was consumed, and the current distortion also stopped changing. From this subtraction, it is clear that the change in the distortion response is a specific set of peaks which are closely linked to the availability of O_2_. 

Thus the advance of the current distortion phase appears to be related to the lack of aerobic respiration/onset of fermentation. A hint regarding its origin comes from the ρ− strain, which does not have a functioning mitochondrial electron transport chain (ETC) due to a partial deletion of its mitochondrial DNA. To confirm the connection between the ETC and the distortion response, ρ+ yeast treated with antimycin, a specific inhibitor of the ETC, were also investigated. Once again, the distortion response was phase advanced in a manner identical to the ρ− strain/fermenting ρ+ strain (Figure 3(c,d)).

Electrochemical reactions at the electrodes may also influence yeast metabolism, but two factors should be considered: (1) the electric field is only applied for a brief period out of the 2 min window between measurements, so any occurring electrochemistry is relatively small and (2) the most important predictor of harmonic response is whether or not the yeast is consuming oxygen (not the oxygen concentration in the buffer). This is especially apparent at the beginning of the ρ+ yeast experiment (Figure 3(c)), where the phase shift is small. It is possible that the electrochemistry may only affect ρ+ yeast and not ρ− yeast and that furthermore this effect coincidentally saturates at the same time the buffer runs out of oxygen. However, this possibility seems unlikely.

These above pieces of evidence strongly suggest the distortion response phase shift is directly related to the metabolic state of the yeast. In particular, the upregulation of fermentation (*i.e*., cessation of aerobic respiration) creates a specific series of peaks which manifests as a phase advance in the current distortion response. 

### 3.1. Model of Yeast-Electrode Interaction

**Figure 4:**
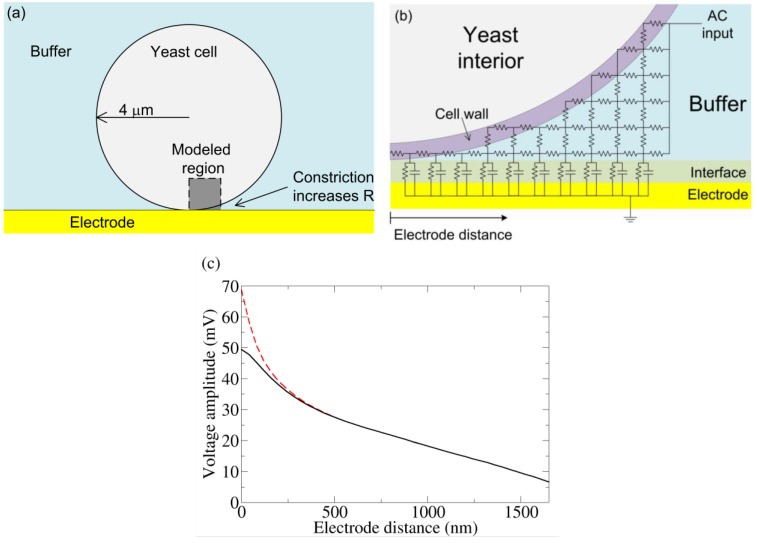
**(a)** Basic model of a yeast cell touching an electrode. Near the bottom of the cell, the volume of conducting buffer is constricted, increasing the resistance underneath. **(b)** A crude schematic of the gap region modeled by an RC circuit. The actual simulation has many more elements. **(c) **From the simulation in (b) and a coarse grain simulation which modeled the entire yeast/electrode system, the voltage drop is measured from the top of the yeast cell to varying positions along the electrode. The “Electrode distance” is measured along the surface of the electrode starting from the point of yeast-electrode contact and is shown in (b). Cell wall conductivities of 50 μS/cm (dashed) and 500 μS/cm (solid) were both simulated. AC voltage conditions: 0.8 V RMS (at the top of the cell), 1 kHz sine wave. We note that without the voltage drop across the interface, this voltage condition would induce electroporation. However, much of the applied voltage is dropped across the electrode interface, and electroporation is unlikely.

To better understand why the yeast dependent harmonics originate at the electrode-interfacial region, we created a simple model of the yeast-electrode system (Figure 4(a)). In Figure 2(a), it is seen that ~1.5 Vpp (1 kHz) is applied across the outer electrodes, but only ~0.1 Vpp is dropped across the sense electrodes. Accounting for the geometry of the electrodes [23], the voltage dropped across the bulk suspension can be calculated as ~0.2 Vpp. By Kirchoff’s voltage law, the rest of the applied voltage (~1.3 Vpp) must be dropped across the outer electrode interfacial region.

Thus the applied sinusoidal voltage across the two outer electrodes is largely dropped at the electrode interface. This is due to the interfacial capacitance. However, when a cell approaches an outer electrode, the cell-electrode constriction increases the charging resistance near the gap, blocking the electrode from charging underneath the cell. This reduces the voltage at the interface, allowing some of the voltage to be dropped across the cell.

To determine the voltage drop across a yeast cell under the experimental conditions used (see below), the system was quantitatively modeled using a 2-D RC network. The outer shell of yeast is composed of an inner insulating plasma membrane and an outer cell wall (conductivity 50 to 500 μS/cm) [24,25]. The yeast cell was modeled as an 8 μm diameter sphere surrounded by a 100 nm thick resistive cell wall. The impedance due to the plasma membrane capacitance (1.1 μF/cm^2^) [24] is large and can be ignored in this frequency range (1 kHz). The suspension media was modeled as a conducting medium (phosphate-buffered saline; 137 mM NaCl, 2.7 mM KCl, 8.1 mM Na_2_HPO_4_, 1.76 mM KH_2_PO_4_; conductivity 15 mS/cm). The electrode-electrolyte interface was modeled as a resistance (50 Ω-cm^2^) and capacitance (220 μF/cm^2^) in parallel, with these values directly measured from the experiment. The resistance accounts for interface dissipation and the capacitance represents the stored charge of the double layer [26]. The Debye length for the buffer is 0.8 nm [27]. Thus, at 100 nm, the Helmholz potential has been attenuated by a factor of 10^−6^. This attenuated Helmholtz potential introduces a small transmembrane potential and is unlikely to play a biological role. It is therefore not considered further. The gold electrodes used in the experiments have a very high conductivity, so the electrode thickness can be ignored in the simulation. The capacitance of the gold wire used in the experiment is larger than expected as compared to other prepared gold surfaces (30 μF/cm^2^). This may be due to the roughness of the gold wire, which would increase the interfacial capacitance. To model the gap, a 40 nm grid was used over a 1.7 μm square region with one corner starting at the bottom of the cell (see Figure 4(a)). Due to the small size scale involved and the high media conductivity, the right side of the gap region could be taken as a constant potential surface (Figure 4(b)); the validity of this approximation was confirmed by a coarse grain simulation (400 nm grid) of the entire yeast cell-electrode system in a 24 μm by 52 μm chamber. At the very bottom of the cell, where the gap is less than the grid spacing, the resistance for each grid element was taken as the parallel combination of cell wall and buffer, the resistances of each being calculated by their respective volume fraction. To investigate the validity of this estimation, the cell wall conductivity was varied from 50 to 500 μS/cm (which is within the reported range of cell wall conductivity).

Figure 4(c) shows the resulting change in voltage drop across the electrode interface as a function of distance away from the zero-gap region. As expected, the electrode capacitance cannot charge fully. The voltage not dropped across the interface is dropped across the cell (confirmed by the coarse grain simulation). Changing the cell wall resistivity does change the potential near the zero gap area, but plays little role beyond 200 nm, indicating any error due to the gap-element’s resistance estimation is unimportant beyond this distance.

From the simulation, we find a greater than 20 mV drop occurs over a 2 μm^2^ patch of a yeast cell facing the electrode. In a simple single shell model of yeast, this potential would be preferentially dropped across the plasma membrane [28]. Although more complicated modeling (especially at higher frequencies) show voltage drops across internal membranes as well [28], we assume a predominant drop across the plasma membrane. In any case this voltage is sufficient to trigger conformation change in voltage-biased electrogenic proteins. In contrast, for a cell away from the electrode, the maximum potential drop across the cell is only 2 mV, which is unlikely to influence membrane protein. We also note that the creation of persistent electrically conducting pores requires >150 mV drop across a membrane [29], a condition not found in this simulation.

### 3.2. Molecular Origin of the Harmonic Response

In summary, we have found respiration dependent harmonic generation of yeast near an electrode interface. A simple computer simulation has also found an enhanced voltage drop across the plasma membrane of yeast near the interface. The question now is: how could mitochondrial activity couple into the electrical harmonic generation?

To address this, we now discuss the potential biological origins of the distortion current. We note the explanation at this point is necessarily hypothetical. Prior work has shown that external electric fields can couple to membrane pumps [9,10], shifting the pump equilibrium towards the conformation with the most favorable charge distribution [8]. For this shift in conformation to occur, the electric field must cross a threshold field, which is set by the free energy barrier to conformational change. When crossed, all the affected pumps will change conformation together. The relatively small applied field applied here would not change overall activity as much as synchronize the existing activity. This synchronized conformation change necessarily involves charge motion (or else it would not couple to the applied field). A number of factors may allow this synchronized activity to be detected. The charge motion of the membrane proteins, while small, would be amplified by the α dispersion—the enhanced dielectric constant found at low frequencies for cellular or colloidal suspension [30]. Such an enhancement would not be found in patch clamp measurements due to the geometry requirements of the α dispersion. This effect could enhance the dielectric constant by 2–3 orders of magnitude [30]. If the activated membrane proteins are voltage dependent ion channels, the charge motion associated with the applied electric field would be further enhanced (due to the electric field driven ion motion through the channel). Quantitative calculation of these effects requires knowledge of the number of proteins near the electrode, which depends on both the concentration of protein within the membrane and the number of yeast near the electrode. The yeast concentration near the electrode may be considerably enhanced due to dielectrophoresis [31]. Increasing the number of yeast near the electrode could dramatically increase measured distortion current and would provide amplification unavailable to traditional patch clamp measurements. However, detailed measurement of the dielectrophoretic effect is a significant endeavor and the subject of ongoing investigation.

These mechanisms would allow membrane protein activity to be probed by harmonic response. For this to be a viable explanation, the fermentation state should drive some membrane proteins to act in a more nonlinear way. One route for harmonic generation stems from proteins in the plasma membrane. Aerobic metabolic activity requires the activity of numerous plasma membrane pumps, including the H+-ATPase (which accounts for 50% of the plasma membrane protein [11]). The fermentation state may drive these proteins into a nonlinear regime. Another possibility stems from the location of mitochondria in the yeast. The yeast used here are in the late log phase of growth, where the mitochondria localize next to the plasma membrane and form an extended, interconnected network with many cristae (folds of the mitochondria surface) that greatly increase the mitochondrial surface area [20,32]. These factors may promote coupling of external electric fields to mitochondrial membrane proteins via interfacial polarization.

## 4. Conclusions

Yeast near a current injection electrode experience an enhanced voltage drop due to the inability of the local electrode capacitance to charge quickly. This enhanced voltage drop allows detection of the yeast displacement current response, the nonlinear component of which is correlated to the degree of mitochondrial ETC activity. This ability may prove useful in the detection of mitochondrial anomalies in whole cells, which has been correlated with the metabolic syndrome [33] (associated with an increased risk of stroke and heart disease) and the up-regulated anaerobic metabolism of many types of cancer (Warburgh effect) [34].
